# Ultrafast Dynamics of rsCherryRev in Comparison with mCherry Reflecting Its Reversive Switchability

**DOI:** 10.1021/acs.jpcb.6c02790

**Published:** 2026-08-13

**Authors:** Yu-Sheng Tsai, Sheng-Ting Hung, Chuang-Yu Lin, Ai Hsu, Bo-Ting Chen, Takayoshi Kobayashi, Atsushi Yabushita

**Affiliations:** † Department of Electrophysics, 34882 National Yang Ming Chiao Tung University, Hsinchu 300, Taiwan; ‡ Department of Physics, National Sun Yat-Sen University, Kaohsiung 80424, Taiwan; § Department of Biomedical Science and Environmental Biology, 38023 Kaohsiung Medical University, Kaohsiung 80708, Taiwan; ∥ Research Institute of Engineering, Kanagawa University, Yokohama, Kanagawa 221-8686, Japan

## Abstract

Ultrafast dynamics of a red fluorescence protein mCherry and its reversibly switchable mutant rsCherryRev was studied by using a 10 fs visible pulse laser and fastscan transient absorption spectroscopy system. This mutation of mCherry for rsCherryRev has caused ultrafast relaxation with lifetimes of ∼0.3 ps and ∼3 ps much faster than the lifetime of mCherry (∼0.5 ns). The ultrashort lifetimes of rsCherryRev are considered to reflect the initial structural change of the chromophore during photoisomerization. The ultrashort duration of 10 fs laser pulse also visualizes that the C=C stretching mode intensity increases at ∼0.7 ps delay, reflecting the timing of photoisomerization in rsCherryRev.

## Graphical abstract

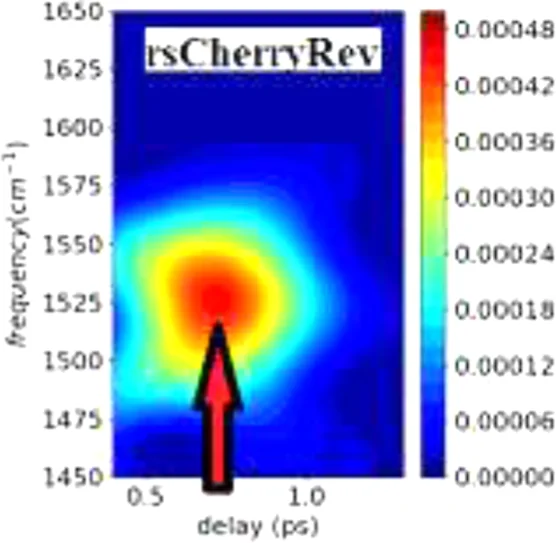

## 1 Introduction

Green fluorescent protein (GFP) is a protein isolated from the jellyfish Aequorea Victoria. GFP research began in the early 1960s when Shimomura et al.^1^ has discovered it while studying the photoprotein aequorin, and its reaction dynamics has been studied widely up to the subpicosecond region.^2–5^ GFP has the unique property of absorbing blue light (approximately 395 nm) or ultraviolet light and emitting green light (approximately 509 nm). This fluorescence is notable because it is expressed solely through the protein’s own amino acid sequence, without the need for external cofactors. In the 1990s, Chalfie et al. introduced the GFP gene into other organisms and demonstrated in vivo fluorescence.^6^ This led to the rapid adoption of GFP as a versatile visualization tool in life science research. Thus, GFP is used in a wide range of fields, including gene expression analysis for visualization of promoter activity,^6^ protein localization analysis for observation of intracellular dynamics by fusing with target proteins,^7,8^ cell lineage tracing for long-term observation in developmental biology and neuroscience,^9,10^ and fluorescence resonance energy transfer (FRET) sensors for real-time measurement of calcium concentration and enzyme activity.^11,12^

As GFP became more widely used, many mutants were developed with improved fluorescence intensity, maturation rate, and pH stability. In particular, research by Tsien et al. led to the development of fluorescent proteins with different fluorescence wavelengths, such as blue (BFP), cyan (CFP), and yellow (YFP), enabling multicolor imaging.^13^

Another FP family is the one including a red FP of DsRed, which is derived from the sea anemone Discosoma sp.^14^ DsRed has the following practical problems: strong tetramer formation (not suitable for use as a fusion protein), slow maturation, spectral heterogeneity due to the accumulation of green intermediates, and cytotoxicity. These limitations directly motivated the development of improved red FP of mFruit, which Tsien et al. has developed using DsRed.^15^ Compared with DsRed, mFruit has the following advantages: fully monomeric (suitable for fusion protein, FRET, and subcellular localization analysis), fast maturation, color diversification (orange to far-red), reduced cytotoxicity, and improved pH and photostability.

mCherry is most widely used in the group of mFruit because of its useful characteristics including rapid maturation speed, high photostability, and red-shifted emission wavelength. Longer-wavelength emission suffers from less scattering effect in thick tissue samples, which is preferable for *in vivo* imaging applications.

The diffraction barrier in fluorescence microscopy at low light intensities was reported to be able to overcome by using a reversibly photoswitchable protein via super-resolution fluorescence microscopy called reversible saturable optical fluorescence transitions (RESOLFT).^16^ Using mCherry, Stiel et al. have developed the reversibly switchable FP (RSFP) with the following mutations on mCherry-Glu^144^Val, Ile^161^Ser, Val^177^Phe, and Lys^178^Trp.^17^ This mutant is called rsCherry, which emits at 610 nm under excitation at ∼550 nm. This mutant is a positive-type RSFP: Irradiation at ∼550 nm converts it from the fluorescent off-state to the on-state, whereas irradiation at ∼450 nm converts it from the on-state to the off-state. During the development of rsCherry, it was found that the Gln^163^ Met mutation on rsCherry reversed the photoswitching wavelength effects.^17^ Figure 1a shows a schematic figure for the photocycle of rsCherryRev where the on-state chromophore adopts a *cis*-conformation and the off-state chromophore a *trans*-conformation. For application of RESOLFT, the reversed switching wavelengths are needed, and the mutant was optimized by Stiel et al. with the following mutations on mCherry-Glu^144^Val, Ser^146^Cys, Ile^161^Ser, Gln^163^ Met, and Val^177^Phe.^17^ This mutant is called rsCherryRev in which lights of ∼450 nm build up the on-state, while irradiation with ∼550 nm largely depopulates the fluorescent states and falls in the category of negative-type RSFPs. As most RSFPs are negative-type, we focus our discussion on them.

**Figure 1:**
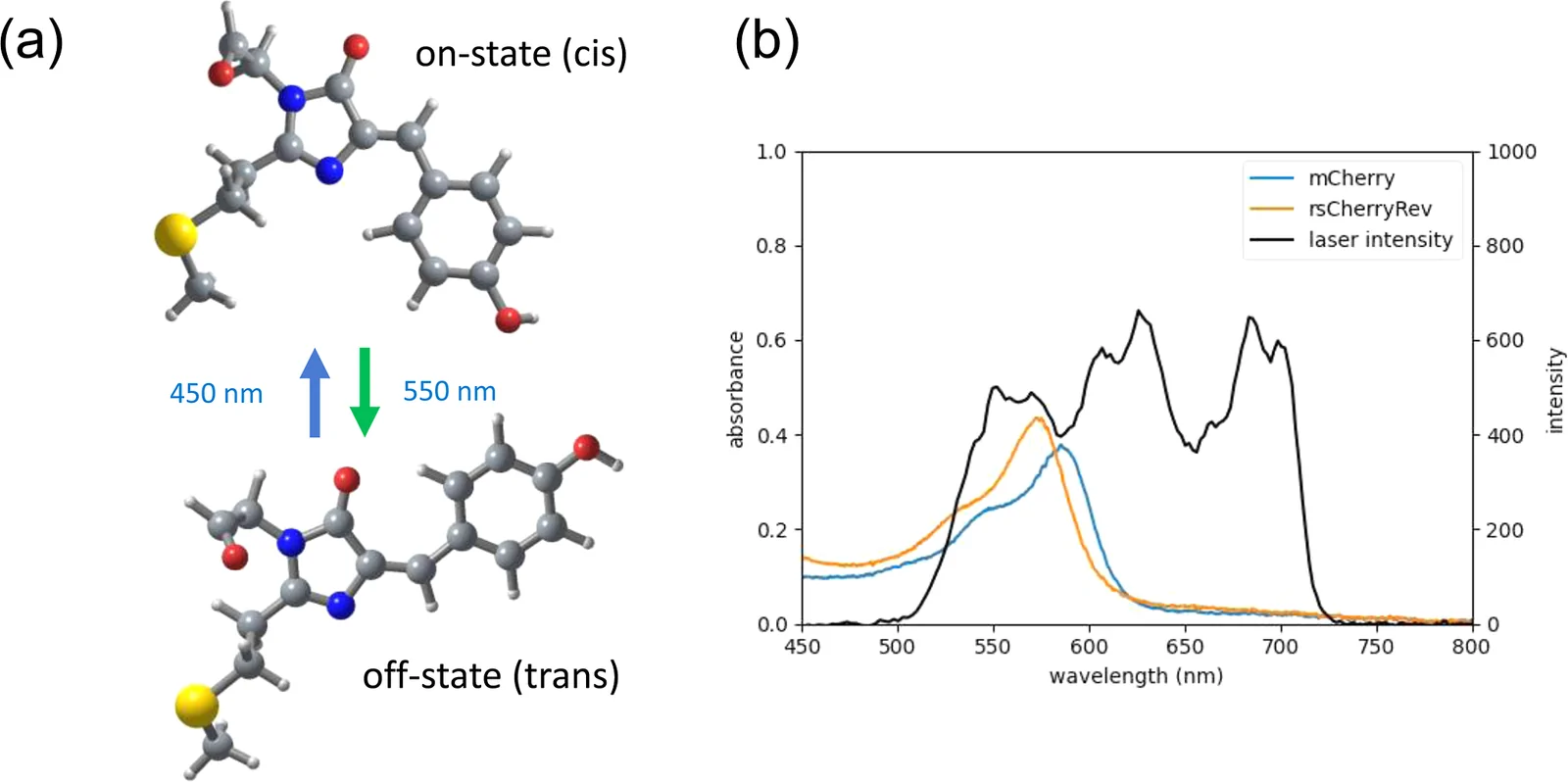
(a) Schematic figure for the photocycle of rsCherryRev shown with switching wavelengths and chromophore structural changes. (b) Laser spectrum (black curve) and absorption spectra of mCherry (blue curve) and rsCherryRev (orange curve)*.*

The chromophore in RSFPs is governed by a complex interplay of cis–trans isomerization, protonation and deprotonation, and structural constraints imposed by the environment. The photoswitching of Dronpa, a green RSFP, originates from a cis–trans isomerization of the chromophore accompanied by rearrangement of four nearby amino acids on the β-barrel, and the different cis- and the trans-cavity affects the protonation equilibria of the chromophore.^18^ The structural rearrangement of the β-barrel introduced by 1–2 mutations at Val^157^ and/or Met^159^ in Dronpa results in a reduced energy barrier for the blue light-driven cis–trans isomerization of the chromophore and significantly increased photoswitching speed.^19,20^ Multimerization of Dronpa variants was demonstrated to affect their on/off switching kinetics due to the reduced flexibility of the β-barrel.^21–23^ The photoswitching rates of the chromophore dictate the single-molecule fluorescence on- and off-time, which are one of the key parameters to single-molecule localization microscopy.^24^ Though the mechanistic insights on the photoswitching of the chromophore of RSFPs are of great interest to advance the rational development of RSFPs, there are limited ultrafast studies on RSFPs. The off-to-on photoswitching of Dronpa and its variant, Dronpa-2, was concluded to occur before the ground-state proton transfer (GSPT) using time-resolved fluorescence, ultrafast transient absorption (TA), and time-resolved infrared (TRIR) spectroscopy.^25^ The photoswitching of rsEGFP2, engineered from wtGFP, was characterized across a wide range of time scales through quantum chemistry simulations and ultrafast spectroscopy techniques. Upon 400 nm photoexcitation, it was recently observed that the off-state rsEGFP2 undergoes photoisomerization through volume-efficient “Hula-Twist” within a spatially constrained β-barrel as early as 300 fs.^26^ The chromophore then relaxes into two conformers in 3 ps, and the planar trans conformer returns back to the off state in 5.5 ps, while the twisted conformer evolves to the *cis*-neutral state in 117 ps.^25^ The chromophore resides entirely in the *cis*-neutral conformation in 10 ns, followed by GSPT with time constants at ∼67 μs and 2 ms, and the β-barrel restructures to accommodate the *cis*-chromophore throughout these chromophore relaxation processes.^25^ Despite the growing interest in the mechanistic studies on RSFPs, ultrafast investigations into red RSFPs remain lacking.

To elucidate the mechanism that differentiates the function of mCherry and rsCherryRev, we have performed ultrafast TA spectroscopy for the two samples of mCherry and rsCherryRev using a 10 fs visible pulse laser. The pulse duration as short as 10 fs enables us to observe signal modulation caused by real-time motion of molecular vibration, and short-time Fourier transform (STFT) analysis of the observed signal visualizes vibrational dynamics reflecting the molecular structure change during the photoreaction.

## 2 Methods

Pure mCherry and rsCherryRev proteins were prepared using a protocol similar to that reported in our previous work^27^ with a different bacterial strain. The plasmid of pBAD_mCherry obtained from Addgene (catalog no. 187387) underwent a point mutation M83L (according to the numbering in the reference) to convert into the commonly used mCherry DNA.^15^ The mutated plasmid was then subjected to the N-terminal conjugation with 6*x*-His tag for further protein extraction. The final product was confirmed to have the same sequence as mCherry reported in the literature^15^ and is referred to mCherry throughout this article. The mCherry plasmid was further mutated to rsCherryRev according to the mutations reported in the literature^17^ using a Q5 Site-Directed Mutagenesis Kit (New England Biolabs), and the sequence of the final product was also confirmed to be identical as that in the literature.^17^

Competent *E. coli* (ECOS 21 Competent Cells [BL21­(DE3)]) were transformed with the plasmid encoding an FP DNA by a conventional heat-shock protocol. 2–5 μL of DNA (∼80 ng/mL concentrations) was gently pipetted into ∼50 μL of competent cells (Invitrogen). The cells were kept on ice for 20 min, heat-shocked at 42 °C for 45 s, followed by growing in an antibiotic-free medium for 45 min. Subsequently, the cells (∼25–50 μL per plate) were plated onto ampicillin-containing LB agar plates with l-arabinose and incubated at 37 °C overnight. Colored colonies were then picked from these plates and grown in the LB broth containing ampicillin at 37 °C and 180 rpm overnight. The following day, 500 μl of overnight culture was transferred into 50 mL of LB broth containing 50 μL of ampicillin (100 mg/mL) and then grown at 37 °C for 4 h, followed by the addition of 5 mL of 20% l-arabinose to initiate expression. At this stage, the incubation temperature was lowered to 28 °C for 24 h to slow bacterial growth and facilitate protein folding and chromophore maturation.

The induced cell cultures were spun down at 6000 rpm, 4 °C, for 15 min, and the cell pellet was stored at −20 °C. Bacterial protein extraction reagent (BugBuster, Merck) was used with lysozyme to lyse cells. Cells were lysed at 37 °C for 1 h and then underwent disruption using a cell disruptor. The solution was then spun down at 12000 rpm and 4 °C for 15 min to separate the FP from the cellular debris. The supernatant was then mixed with equilibration buffer of 10 mM imidazole and loaded into a purification column (Thermo scientific) and left at 4 °C overnight. The column was then washed with 10 mM imidazole, followed by a subsequent wash using 25 mM imidazole, and then the FP was eluted with 250 mM imidazole. After purification, a centrifugal filter (10 kDa; Amicon) was used to concentrate the protein, and the imidazole was removed during this process through excess PBS wash.

For sample solutions of mCherry and rsCherryRev, we have performed TA spectroscopy using 10 fs visible broadband laser pulse in a fastscan mode of the TA spectroscopy system. To photoswitch rsCherryRev, two cw lasers with wavelengths of 450 and 532 nm were used at intensities 13 W/cm^2^ and 35 W/cm^2^, respectively. These wavelengths are consistent with the literature that demonstrated the switchability of rsCherryRev, in which the excitation wavelengths were chosen at 450 ± 20 nm and 550 ± 20 nm.^17^ To increase the photoswitching efficiency, the switching light intensities adopted in this study are moderately higher than those used in the literature (∼2 W/cm^2^ for 450 nm and ∼4 W/cm^2^ for 550 nm),^17^ but they are still orders of magnitude lower than those used in RESOLFT with a rsCherryRev mutant.^28^ Preparation methods for the sample solutions and optical systems are described below.

Samples of mCherry and rsCherryRev used in the TA spectroscopy measurements were prepared using the same protocol reported in our previous work.^27^ To measure the samples in stable pH conditions of pH = 7.5, these two FPs were prepared in 100 mM Tris-TCl buffer with 150 mM NaCl. At room temperature (297 K), each of these sample solutions was stored in a screw-capped quartz glass cell (S15-UV-2, GL Science Inc.), respectively. The quartz cell has an internal optical length of 2 mm, and the wall thickness is 1.25 mm each for the front and back walls. Another quartz cell was intentionally broken into two pieces to have its front wall. As described later, the front wall of the glass cell was used to evaluate material chirp which the wall of the quartz glass cell gives to the 10 fs visible broadband laser pulse.

As a light source of the 10 fs visible broadband pulse laser, we have used a Ti/sapphire regenerative amplifier (Legend USP-5K-HE, Coherent Inc.) which generates near-infrared (NIR) pulse (center wavelength 800 nm, pulse energy 0.5 mJ, repetition rate 5 kHz, and pulse duration 35 fs). The NIR pulse was separated into two copy pulses to generate near-ultraviolet (NUV) pulse and broadband visible pulse by second harmonic generation (SHG) in a 0.1 mm-thick β-BaB_2_O_4_ (BBO) crystal and self-phase modulation in a 2 mm-thick sapphire plate, respectively. The NIR component was removed from the broadband visible pulse whose pulse duration was compressed by using a chirped mirror pair. Then, the NUV pulse and the broadband visible pulse were focused into a 1 mm-thick BBO crystal to be used as a pump pulse and a seed pulse, respectively, of noncollinear optical amplifier (NOPA)^29–31^ to amplify the seed pulse. These two pulses, the remainder of the pump pulse and the amplified seed pulse, were reflected by a spherical mirror to amplify the amplified seed pulse again in the same BBO crystal. Pulse duration of the output pulse, which was amplified twice in NOPA, is tunable by a homemade pulse compressor system adjusting chirp.

The output pulse of NOPA with vertical polarization transmitted through a beam sampler made of a quartz glass plate to generate two copy pulses with pulse energies of 18 and 2 μJ to be used as a pump pulse and a probe pulse, respectively, in the pump–probe measurement for TA spectroscopy. The pump and probe pulse transmit the beam sampler once for each in the pump–probe measurement system, which provides the same chirp to these two pulses. The pulse duration of these two (pump and probe) pulses was set to have the shortest pulse duration at the position of the sample solution (after transmitting through the front wall of the quartz glass cell) as follows.

For the pulse characterization, 10μm-thick BBO crystal was used to generate the sum frequency generation (SFG) of the pump pulse and the probe pulse for SHG frequency-resolved optical gating (FROG).^32^ In front of the BBO crystal, one front wall of the quartz glass cell was inserted in the optical path. Thus, the pulse characterization result reflects that at the position of the sample solution in the glass cell. The pulse compressor system was adjusted to get the highest intensity of the SFG signal and the pulse train retrieved from the measured SHG-FROG trace has confirmed that the pulse duration is 10 fs for each of those two (pump and probe) pulses. The detail of the optical system to generate the 10 fs visible broadband laser pulse was described elsewhere.^33^ All of the experiments were conducted at room temperature (293 ± 1 K).

## 3 Results and Discussion

Figure 1b shows the laser spectrum of the 10 fs visible broadband laser pulse (black curve) and the absorption spectra of the sample solutions for mCherry (blue curve) and rsCherryRev (orange curve). The absorbance of rsCherryRev indicates its concentration to be ∼25 μM (calculated based on the extinction coefficient of rsCherryRev^34^ and the 2 mm optical length). The monomeric rsCherryRev is 5 mutations away from mCherry, and only Glu^144^Val mutation is an outward residue. There is a possibility that rsCherryRev remains monomeric in our experimental condition, as mCherry is known to retain its monomericity up to at least 25 μM.^35^ The absence of scattering noise in the observed TA spectra suggests that the oligomer is not formed in the rsCherryRev sample solution under the experimental condition of the present work.

The sample of rsCherryRev was measured by irradiating the solution with a blue laser diode with a wavelength of 450 nm. The result shows that the laser spectrum overlaps with the absorption bands of the samples at wavelengths shorter than 620 nm. Thus, the measurement result of the TA spectrum at the wavelength longer than 620 nm does not include the electronic ground-state bleaching (GB) signal but reflects induced absorption (IA) and/or stimulated emission (SE) of the electronic excited state.

TA spectra of the samples of mCherry and rsCherryRev were measured with scanning optical delay of the probe pulse from the pump pulse. The delay scan was performed using a stepping motor stage (SGSP26-200­(X), Sigma Koki), which is called step-scan pump–probe spectroscopy. The measured results are shown in Figure 2a,b, respectively.

**Figure 2:**
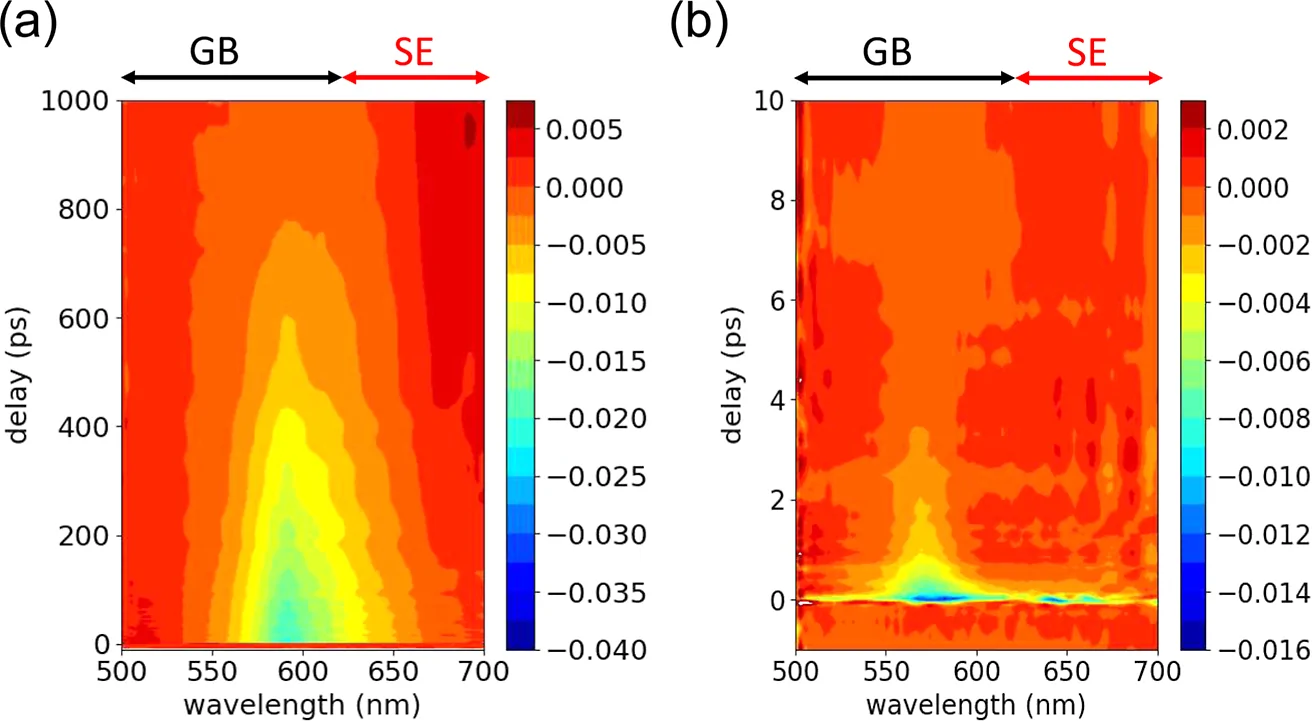
Two-dimensional (2D) view of TA traces measured by a conventional step-scan pump–probe spectroscopy for the samples of (a) mCherry and (b) rsCherryRev. GB (SE) indicates the region where the signal is dominated by that of GB (SE).

The measured step-scan TA traces of the whole probe wavelength region were analyzed by global fitting analysis using an R library TIMP, which is a package for fitting separable nonlinear models in spectroscopy and microscopy.^36^

The decay rate of the mCherry sample was estimated to be *k*_1_ = 2.2 × 10^–3^[ps^–1^] (another lifetime was fixed to be *k*_∞_ = 1 × 10^–9^[ps^–1^], which is for a signal component not decaying in the measured delay region of 1 ns). Decay associated spectra (DAS) corresponding to *k*_1_ and *k*_∞_ are called here as DAS_1_ and DAS_∞_, respectively (see Figure 3a). DAS_1_ has a positive sign signal at a wavelength shorter than 530 nm which can be attributed to IA. A negative sign signal around 590 nm with a tail extending to 680 nm is thought to be dominated by GB because the stationary absorption spectrum of mCherry has a peak around the wavelength of 590 nm (see Figure 1). Considering that the stationary absorption spectrum has a tail to ∼620 nm, the negative sign signal in the region from 620 to 680 nm can be assigned to SE. It estimates the lifetime of the first state after photoexcitation is 450 ps. DAS_∞_ has a positive sign signal with two peaks around 590 and 670 nm. It shows that the second state after photoexcitation is not the electronic ground state having absorption bands of these two peaks.

**Figure 3:**
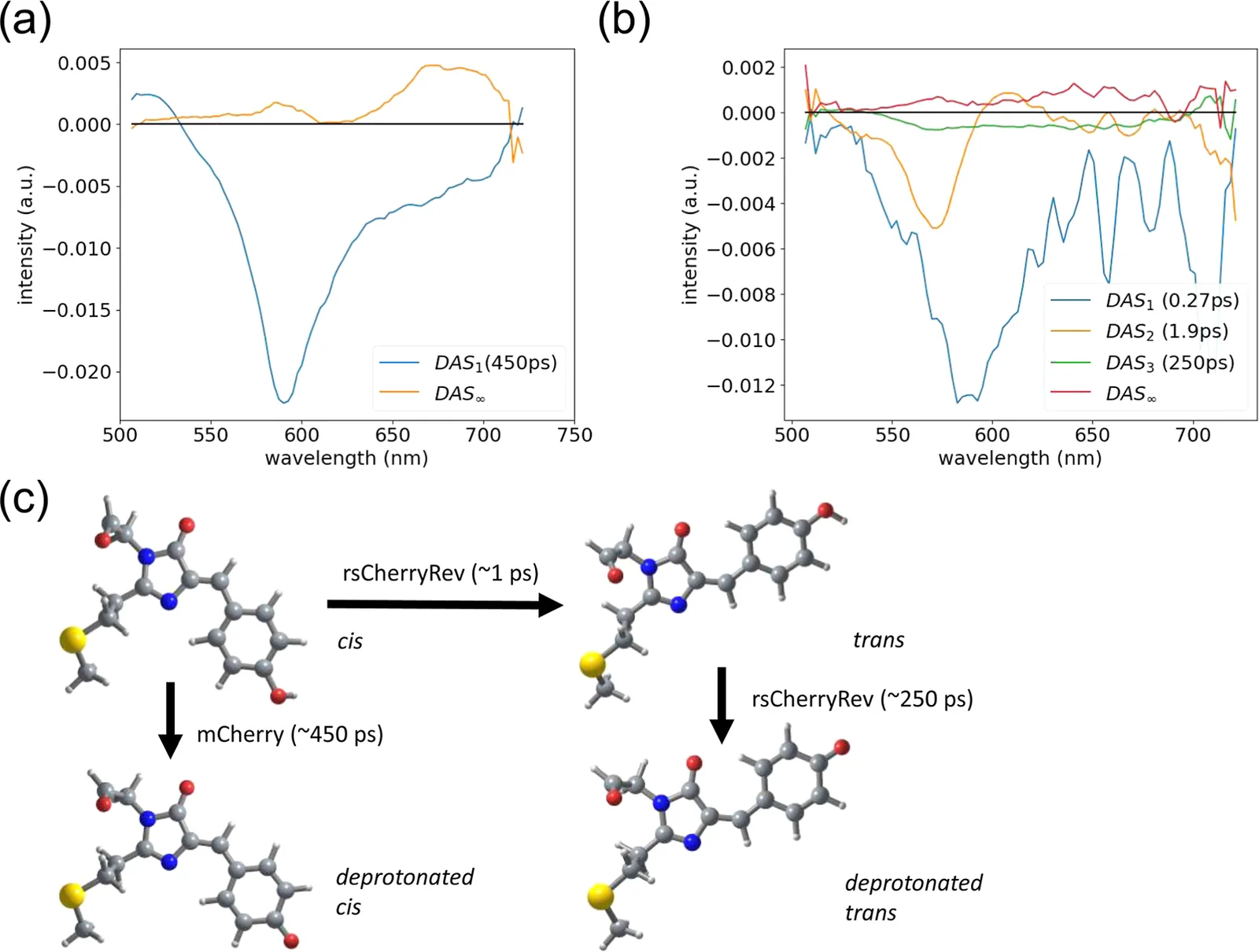
DAS calculated by global fitting analysis of the measured TA traces (see Figure 2) for the samples of (a) mCherry and (b) rsCherryRev. DAS_*i*_ (*i* = 1, 2, 3, ∞) corresponds to a lifetime of τ_*i*_ estimated in the global fitting analysis. (c) Schematic figure showing relaxation pathways of mCherry and rsCherryRev.

Global fitting analysis was also performed for the sample of rsCherryRev estimating the decay rates to be *k*_1_ = 3.7 [ps^–1^], *k*_2_ = 5.2 × 10^–1^[ps^–1^], and *k*_3_ = 4.0 × 10^–3^[ps^–1^] with a fixed one of *k*_∞_ = 1.0 × 10^–9^[ps^–1^]. DAS_1_ has a negative sign observed around 580 nm which can be assigned to GB because of the stationary absorption of rsCherryRev with a peak around the wavelength of 580 nm (see Figures 1 and 3b). In the same way as the mCherry case, the negative-sign signal at wavelength longer than 620 nm is thought to be assigned to SE. DAS_2_ has a negative sign signal around 580 nm and a positive sign signal around 600 nm, which can be assigned to GB and IA, respectively. DAS_3_ and DAS_∞_ have the same spectral shape with the opposite sign to be a negative sign signal and a positive sign signal, respectively. It shows that the intermediate state with the IA signal of DAS_∞_ appears with the rate of *k*_3_, and the intermediate state is not the electronic ground state. Considering the typical time scale of photoisomerization is <1 ps, the decay rate of *k*_3_ is thought to be assigned to deprotonation. Thus, the positive feature of DAS_∞_ can be assigned to the IA signal of the deprotonated trans conformer, which appears with the rate of *k*_3_ being observed as the negative feature of DAS_3_. The decay rates of *k*_1_(∼270fs) and *k*_2_(∼1.9ps) are thought to reflect the initial structural change of the chromophore during photoisomerization. Figure 2b shows that the TA spectral peak shifts to a longer wavelength in this time scale of *k*_2_. To identify whether this spectral shift is caused by the dynamics of on-state, we have performed TA measurement of the rsCherryRev sample irradiating 450 nm laser and 532 nm laser to prepare on-state and off-state, respectively. The result shown in the Supporting Information (see Figure S1) indicates that the TA spectral shift reflects the dynamics of on-state (not observed for off-state).

For further clarification, we have measured the TA signal with a finer delay step size of 3.6 fs using the 10 fs visible pulse laser to temporally resolve molecular vibration. To obtain enough signal-to-noise ratio for the analysis of signal modulation caused by molecular vibration, we have measured those TA signals using the fastscan TA spectroscopy system whose detail was previously reported elsewhere.^37,38^ STFT of the measured fastscan TA trace calculates the temporal change of molecular vibration frequency reflecting the dynamics of molecular structure change after the photoexcitation as shown below.

The measured fastscan TA signals of mCherry and rsCherry samples are shown in Figure 4a,b, respectively, which are averages of 40 scans of data.

**Figure 4:**
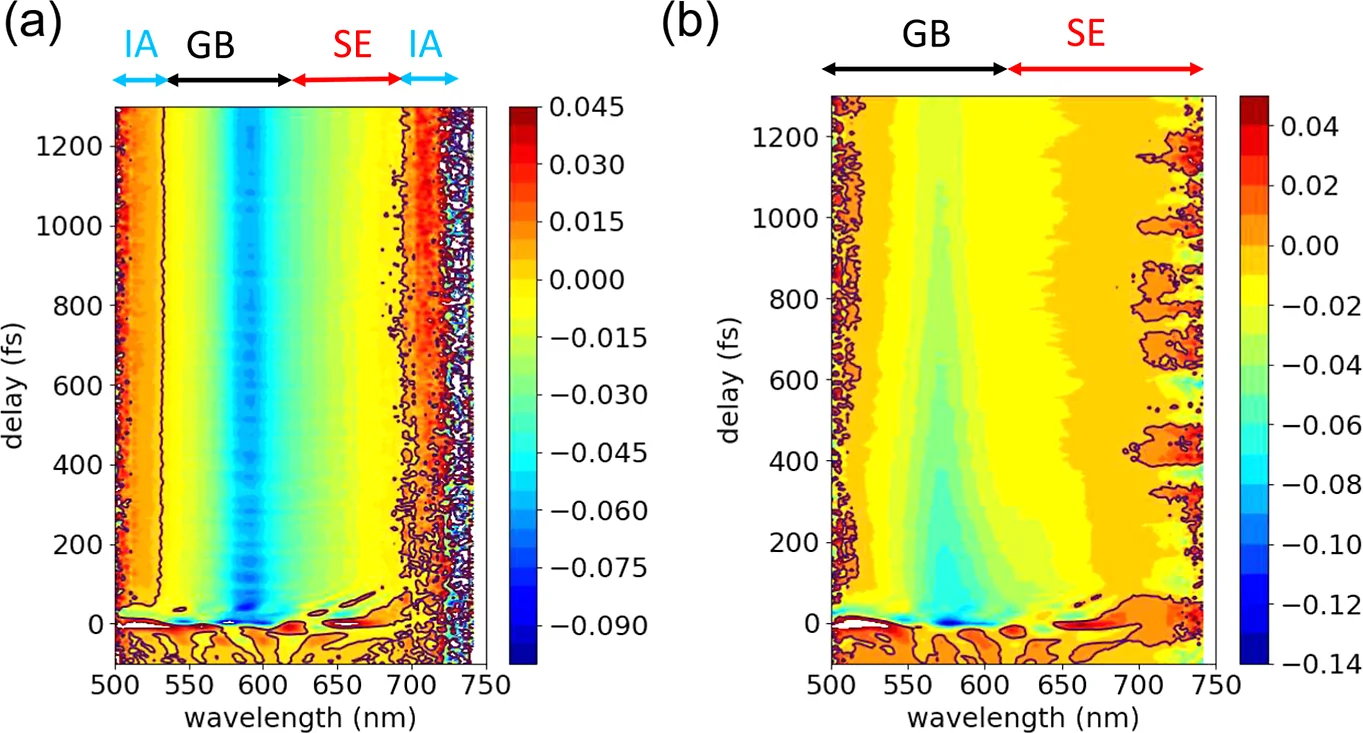
2D view of TA traces measured by a fastscan pump–probe spectroscopy for the samples of (a) mCherry and (b) rsCherryRev. GB, SE, and IA indicate the region where the signal is dominated by that of GB, SE, and IA, respectively.

The Fourier power spectrum of the measured TA trace was calculated for every probe wavelength after applying a high-pass filter to pass frequencies higher than 180 cm^–1^. To avoid noise due to the coherent artifact effect existing around the zero delay region,^39,40^ the delay region of the TA trace used in the Fourier transform analysis was started from 100 fs after photoexcitation. Calculated Fourier power spectra of mCherry and rsCherryRev are shown in Figure 5a,b, respectively.

**Figure 5:**
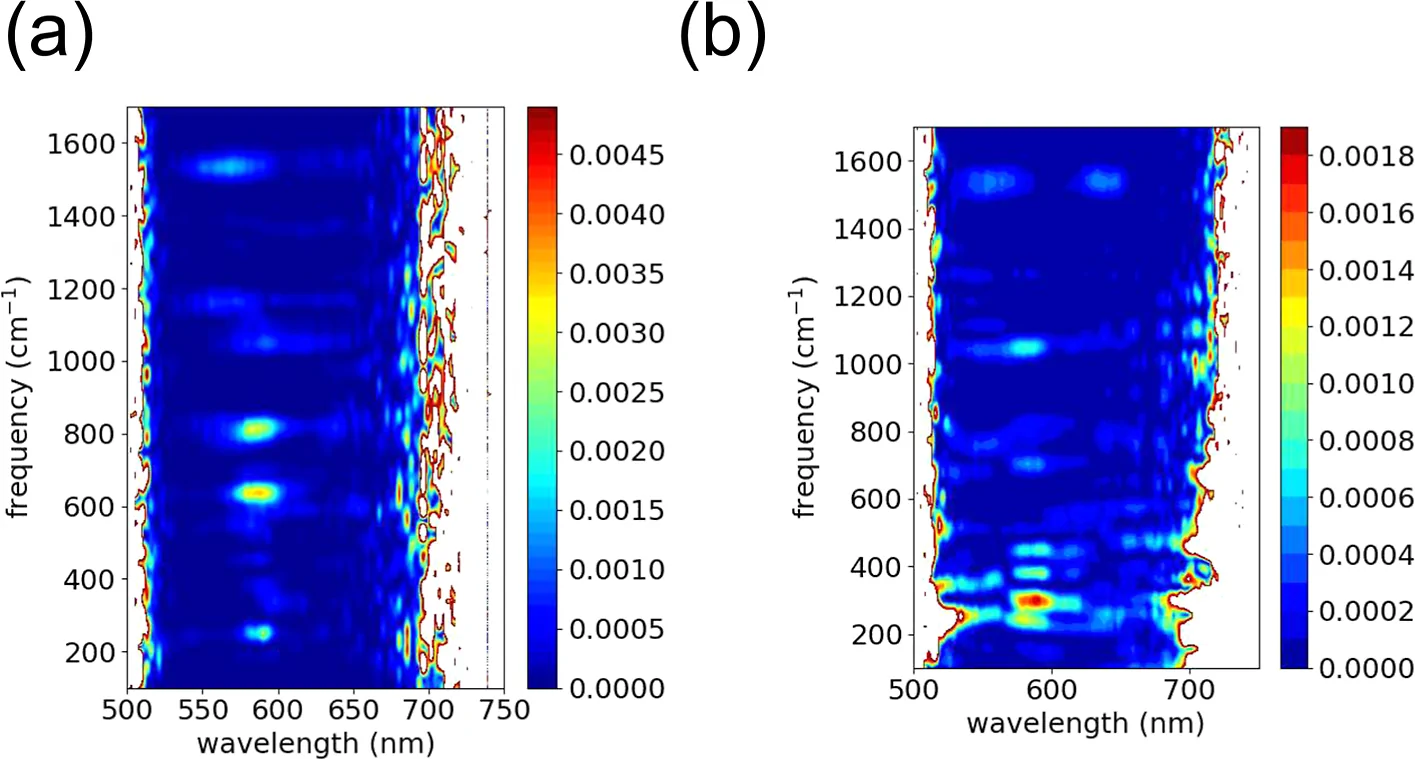
2D view of Fourier power spectra of the measured TA traces (see Figure 4) for the samples of (a) mCherry and (b) rsCherryRev.

The Fourier power spectra of both samples show an intense signal around 590 nm close to the peaks of their absorption bands (see Figures 1 and 5).

Those modes observed around 590 nm are thought to be that of the electronic ground state. The signal observed around 1550 cm^–1^ can be assigned to the C=C stretching mode, ν_C=C_. This ν_C=C_ mode for each sample was observed around 640 nm where the sample has no absorption band (see Figures 1 and 5). Thus, the signal found at 640 nm is thought to be that of the electronic excited state. Using the TA trace probed at 640 nm, the vibrational dynamics of this ν_C=C_ mode was analyzed by STFT calculating the spectrogram trace. In the calculation of the spectrogram trace, the Blackman window function whose full width at half-maximum is 600 fs (i.e., frequency resolution is 56 cm^–1^) was used to identify the dynamics of modes separated by 56 cm^–1^ or larger.

For the discussion of the vibrational dynamics, we have calculated Raman activity spectra of a chromophore which is common in mCherry and rsCherryRev. The calculation was performed using Gaussian 16 software,^41^ the B3LYP method, and a basis set of 6–31+(G)­d. In mCherry, the chromophore exists as the cis conformer, so photoexcitation just results in deprotonation. In rsCherry, the photoexcitation can cause photoisomerization producing the trans isomer not only causing deprotonation. Thus, calculation was performed for cis conformer, deprotonated cis conformer, trans conformer, and deprotonated conformer for the ν_C=C_ mode (see Figure 6).

**Figure 6:**
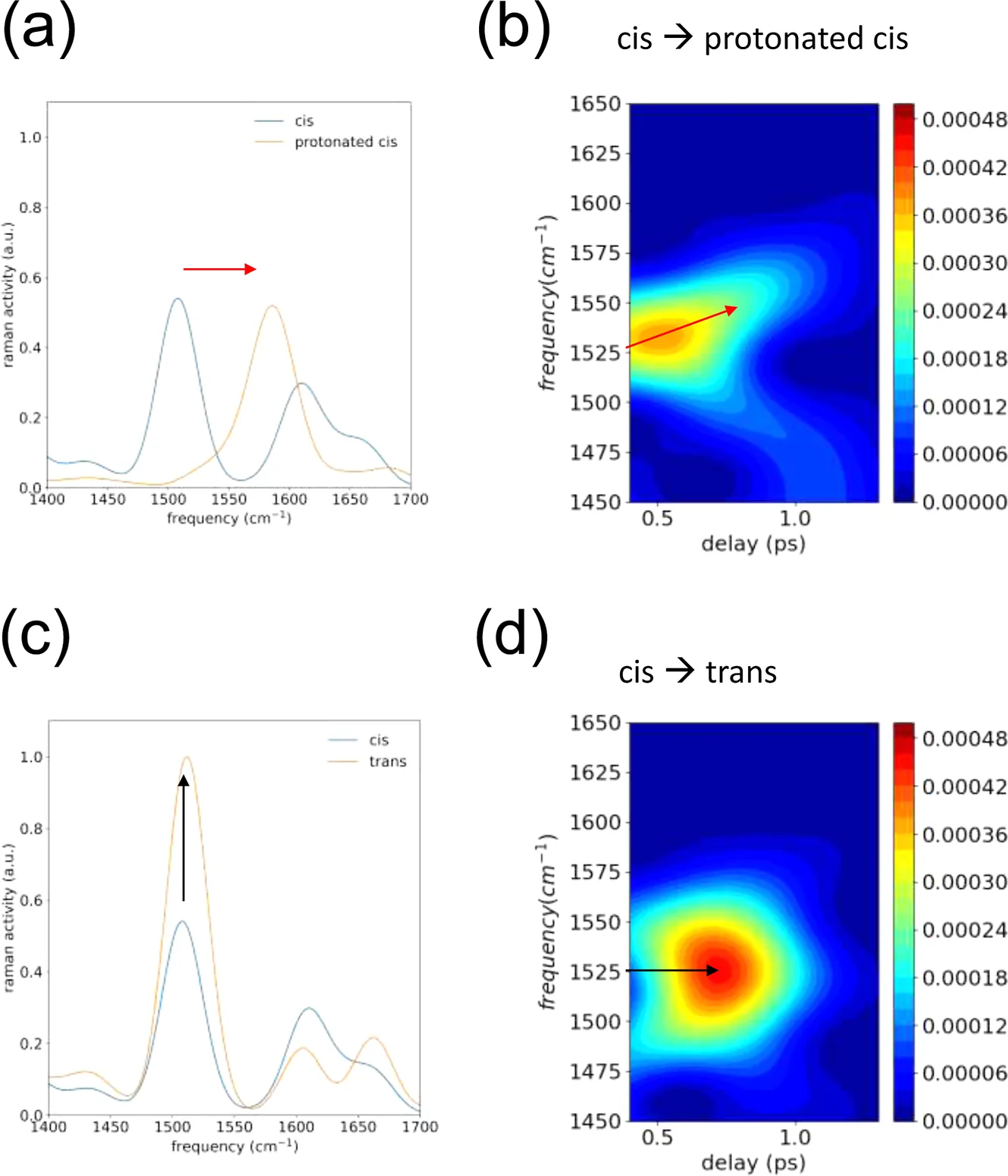
(a,c) Raman activity spectrum calculated by quantum chemical calculation corresponding to (a) deprotonation in mCherry and (c) photoisomerization in rsCherryRev. (b,d) Spectrogram trace calculated by STFT analysis of the TA trace measured at 640 nm where the signal of the electronic excited state dominates for the samples of (b) mCherry and (d) rsCherryRev.

The calculation result shows that deprotonation causes frequency upshift in the cis conformer (see Figure 6a), and photoisomerization from the cis conformer to the trans conformer enhances Raman activity (see Figure 6c).

The TA signal of the mCherry sample probed at 640 nm was used to calculate the spectrogram trace (see Figure 6b). The result shows that the ν_C=C_ mode just decays with a slight upshift of frequency. The upshift of frequency is thought to be reflecting deprotonation (of cis conformer) after photoexcitation. Figure 6c shows the calculated spectrogram of the rsCherryRev sample probed at 640 nm. The intensity of the ν_C=C_ mode has increased to its maximum at 0.73 ps after photoexcitation, indicating that the photoisomerization of rsCherryRev proceeds in 0.73 ps.

## 4 Conclusions

The present work has performed ultrafast TA spectroscopy of mCherry and its reversibly switchable mutant rsCherryRev. Global fitting analysis for the TA traces has estimated the lifetime of mCherry to be 450 ps. The relaxation lifetime of rsCherryRev was found to be 0.27, 1.9, and 250 ps using a 10 fs visible pulse laser and fastscan TA spectroscopy system. For both samples (mCherry and rsCherryRev), DAS corresponding to the TA signal not decaying in the measured delay region of 1 ns was found to have a positive sign signal. It indicates that the longest lifetime (450 ps in mCherry and 250 ps in rsCherryRev) is the transition to an intermediate state (not the electronic ground state), which produces an IA signal with a positive sign. The ultrashort lifetimes of rsCherryRev (0.27 and 1.9 ps) are considered to reflect the initial structural change of the chromophore during photoisomerization.

The ultrashort pulse duration of 10 fs has visualized molecular vibration in the time domain to study vibrational dynamics. STFT analysis of the TA trace probed at 640 nm where the signal originates from the electronic excited state was performed to calculate the spectrogram trace for rsCherryRev. The calculated trace shows an increase of the ν_C=C_ mode at 0.73 ps, indicating that the photoisomerization of rsCherryRev proceeds with this lifetime.

The switchability of rsCherryRev has been said to be caused by photoisomerization, which does not occur in mCherry. The present work has visualized this initial structural change of the rsCherryRev chromophore proceeding in the femtosecond region. Thus, ultrafast TA spectroscopy using a 10 fs laser pulse is expected to provide key information on the excited-state behavior and the relaxation pathways of fluorescent proteins and to understand the difference in the functionality of fluorescence proteins under mutation.

## Supplementary Material



## Notes

Transient absorption spectroscopy and 2D view of TA traces (PDF)
