# Supplementary material for: Ultrafast Dynamics of rsCherryRev in Comparison with mCherry Reflecting Its Reversive Switchability
Source: J Phys Chem B. 2026 Aug 13;130(34):8735–43. doi: 10.1021/acs.jpcb.6c02790 (PMC13528277; doi:10.1021/acs.jpcb.6c02790)
Supplement: Supplementary file 1 [file jp6c02790_si_001.pdf]

Ultrafast dynamics of rsCherryRev in comparison with mCherry reflecting its reversive switchability

Yu-Sheng Tsai[†], *Sheng-Ting Hung*[§,*], *Chuang-Yu Lin*[#], *Ai Hsu*[#], *Bo-Ting Chen*[§], *Takayoshi Kobayashi*[†,‡], *and Atsushi Yabushita*[†,‡,*]

[†]Department of Electrophysics, National Yang Ming Chiao Tung University, Hsinchu 300, Taiwan

[§]Department of Physics, National Sun Yat-Sen University, Kaohsiung 80424, Taiwan

[#]Department of Biomedical Science and Environmental Biology, Kaohsiung Medical University, Kaohsiung 80708, Taiwan

[‡]Research Institute of Engineering, Kanagawa University, Kanagawa, 221-8686, Japan

Contents

Section S1. Transient absorption spectroscopy of the rsCherryRev sample under irradiation of 450 nm laser and 532 nm laser **S2**

Section S1. Transient absorption spectroscopy of the rsCherryRev sample under irradiation of 450 nm laser and 532 nm laser

We have performed transient absorption (TA) spectroscopy of the rsCherryRev sample irradiating 450 nm laser and 532 nm laser to prepare on-state and off-state, respectively. The irradiation intensities for 450 nm laser and 532 nm laser were set to be same as the previous work reported by Stiel et al.[1] The optical delay was scanned from -13.3 ps to 53.2 ps with step size of 0.13 ps. The TA measurement was repeated for ten scans to improve S/N ratio of the signal taking their averages. The measured results are shown in Fig. S1.

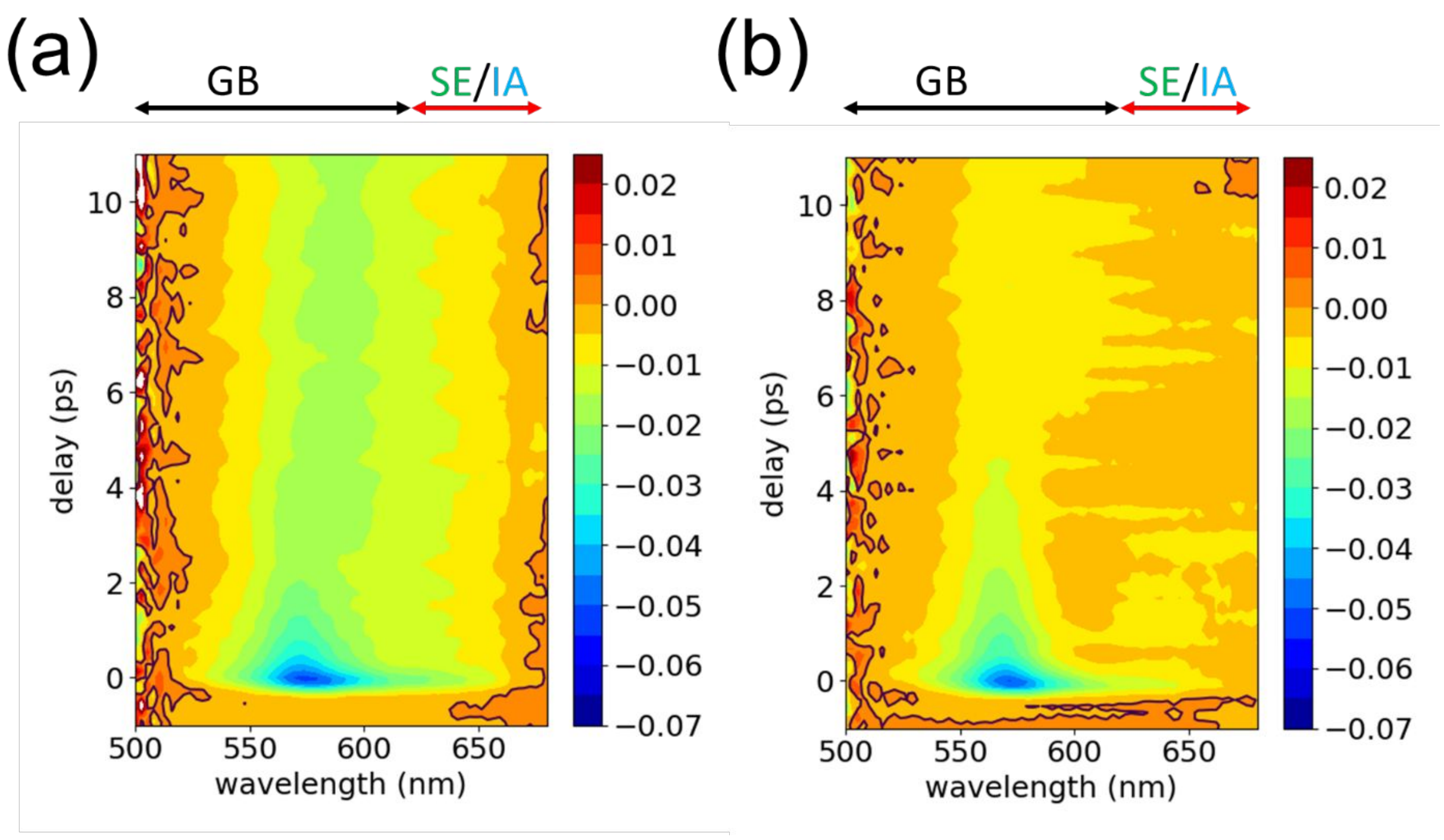

Fig. S1 (a) 2D view of TA traces of the rsCherryRev sample under irradiation of 450 nm laser and (b) 532 nm laser. Black curve shows contour where TA signal was observed to be zero. GB (SE/IA) indicates the region where the signal is dominated by that of GB (SE and IA).

TA spectrum was found to shift for longer wavelength under irradiation of 450 nm producing on-state, and the shift was not clearly observed under irradiation of 532 nm producing off-state. It indicates that the estimated relaxation rate of $k_2(\sim 1.9ps)$ reflects the dynamics of on-state in initial structural change of chromophore during photoisomerization.

REFERENCE

(1) Stiel, A. C.; Andresen, M.; Bock, H.; Hilbert, M.; Schilde, J.; Schönle, A.; Eggeling, C.; Egner, A.; Hell, S. W.; Jakobs, S. Generation of Monomeric Reversibly Switchable Red Fluorescent Proteins for Far-Field Fluorescence Nanoscopy. *Biophys. J.* **2008**, *95* (6), 2989–2997. https://doi.org/10.1529/biophysj.108.130146.
